# Impact of Antimicrobial Resistance of *Pseudomonas aeruginosa* in Urine of Small Companion Animals in Global Context: Comprehensive Analysis

**DOI:** 10.3390/vetsci12020157

**Published:** 2025-02-11

**Authors:** Ana Pereira, Telma de Sousa, Catarina Silva, Gilberto Igrejas, Patrícia Poeta

**Affiliations:** 1 CECAV—Veterinary and Animal Research Centre, University of Trás-os-Montes and Alto Douro, 5000-801 Vila Real, Portugal; anafilipalp@gmail.com; 2 MicroART-Antibiotic Resistance Team, Department of Veterinary Sciences, University of Trás-os Montes and Alto Douro, 5000-801 Vila Real, Portugal; telmaslsousa@hotmail.com (T.d.S.); xusilva2002@gmail.com (C.S.); 3 Department of Genetics and Biotechnology, University of Trás-os-Montes and Alto Douro, 5000-801 Vila Real, Portugal; gigrejas@utad.pt; 4 Functional Genomics and Proteomics Unit, University of Trás-os-Montes and Alto Douro, 5000-801 Vila Real, Portugal; 5 Associated Laboratory for Green Chemistry, University NOVA of Lisbon, 1099-085 Caparica, Portugal; 6 Veterinary and Animal Research Centre, Associate Laboratory for Animal and Veterinary Science (AL4AnimalS), University of Trás-os-Montes and Alto Douro, 5000-801 Vila Real, Portugal

**Keywords:** *Pseudomonas aeruginosa*, urinary tract infections, small animals, resistance patterns, veterinary medicine

## Abstract

The isolation of multidrug-resistant (MDR) bacteria from the urinary tracts of pets is increasingly common, particularly in animals with concurrent health conditions. *Pseudomonas aeruginosa* (PA) is one of the most significant antimicrobial-resistant bacteria affecting cats and dogs within the European Union (EU). This study aims to review the prevalence and antimicrobial resistance patterns of PA isolated from urine samples of small animals globally. This pathogen is known for its opportunistic infections and is a significant concern in veterinary medicine due to its inherent resistance to multiple antibiotics and its ability to acquire additional resistance mechanisms. This review seeks to enhance educational initiatives regarding the management of emerging MDR bacteria.

## Simple Summary

Pets and humans are increasingly being infected with multidrug-resistant bacteria. *Pseudomonas aeruginosa* is a significant challenge in clinic practice due to its resistance to multiple antibiotics. This study aims to review the global prevalence and resistance patterns of *Pseudomonas aeruginosa* in small animals with urinary tract infection in order to improve management strategies for emerging MDR bacteria.

## 1. Introduction

Bacterial urinary tract infections (UTIs) are commonly diagnosed in veterinary medicine and are among the leading causes of antibiotic prescriptions [1,2]. Over the past two decades, there has been a reported increase in MDR bacteria associated with UTIs in companion animals [3]. This trend poses therapeutic challenges and public health concerns, as direct contact between humans and pets facilitates the transmission of MDR bacteria [3,4]. A wide variety of bacterial agents can cause UTIs, with *Escherichia coli* (*E. coli*) being the most frequently isolated pathogen in canines, felines, and even humans [5,6]. Other organisms, such as *Pseudomonas aeruginosa*, are less commonly found in uncomplicated UTIs, but become prominent in animals with recurrent infections and often exhibit multidrug resistance [7,8].

The prevalence and the percentage of resistance of PA to certain antibiotics associated with UTIs in cats and dogs have been poorly characterized in veterinary medicine [9].

Understanding which pathogens are prevalent in pet populations and their resistance patterns allows for more effective treatment options, reduces the risk of zoonotic infections, and facilitates the development of targeted interventions. This knowledge also supports responsible antibiotic use in veterinary medicine, helping to mitigate the growing concern of antibiotic resistance that can impact both animal and human health. By fostering a collaborative approach between veterinarians, pet owners, and public health officials, we can enhance overall community health and ensure safer environments for both pets and their human companions. Thus, the objectives of this review are to summarize the prevalence of PA in UTIs in small animals and to provide a clinical approach to managing these patients.

The classification of antibiotic resistance for PA will be carried out in accordance with Magiorakos et al. [10]. Thus, MDR (multidrug-resistant) bacteria refers to bacteria that demonstrate nonsusceptibility to at least one agent in three or more categories of antimicrobial agents. Bacteria classified as XDR are nonsusceptible to at least one agent in all categories of antimicrobial agents but remain susceptible to two or fewer categories. PDR (pan-drug-resistant) bacteria exhibit nonsusceptibility to all agents in all categories of antimicrobial agents. The term ESKAPE pathogens refers to a group of six bacterial pathogens—*Enterococcus faecium*, *Staphylococcus aureus*, *Klebsiella pneumoniae*, *Acinetobacter baumannii*, *Pseudomonas aeruginosa*, and *Enterobacter* spp.—which pose a significant threat due to their ability to escape the effects of antibiotics. The therapeutic options for infections caused by ESKAPE pathogens are increasingly limited, necessitating ongoing surveillance and the development of new antimicrobial strategies [11].

## 2. Methods

The research documents analyzed in this work were extracted from the Elsevier Scopus and PubMed databases. The search queries (TITLE-ABS-KEY({*Pseudomonas aeruginosa*}), (TITLE-ABS-KEY({antibiotic resistance}), and (TITLE-ABS-KEY({nosocomial infection }) were used in February 2020 for collecting academic documents and patents including “*Pseudomonas aeruginosa*” and/or “antibiotic resistance” and/or “nosocomial infection” terms in the title, abstract, and/or keywords, from the year 2000. Only publications in English were included. The articles from the search were assessed according to document type, language, and inclusion of subject categories. They were further analyzed, and the results were used to write this review. The article selection process was carried out manually, based on information extracted from the databases. No specific software was used to include the articles, although the management of the search and references was facilitated by tools such as Mendeley.

## 3. *Pseudomonas aeruginosa*

PA are opportunistic and ubiquitous Gram-negative bacteria that play a significant role in nosocomial infections [11,12]. This pathogen has the capacity to infect multiple anatomical sites across various animal species, including humans [12,13]. In recent years, there has been an alarming rise in MDR strains of PA [14] attributed to their ability to resist a variety of antibiotics, including aminoglycosides, quinolones, and β-lactams, as well as the emergence of new carbapenemase-producing strains [15]. The urgent need for the development of new antibiotics against these pathogens led to PA being included as an “ESKAPE” pathogen and classified as one of the “high-priority pathogens” by the World Health Organization (WHO) [16,17,18].

### 3.1. Pathogenesis and Virulence Factors

PA is most associated with otitis in dogs; however, due to its opportunistic nature, it can also lead to a wide variety of other infections. Hattab et al. [19], Gómez et al. [13] and Dègi et al. [20] reported higher incidences of this pathogen in ear infections, skin infections, and, to a lesser extent, urinary tract infections. In cats, although infections caused by PA are less common, they predominantly affect the respiratory tract and rarely the urinary tract [21,22].

Understanding host–pathogen interaction is critical for the development of effective therapeutic strategies to control damage to the bladder [23,24]. The pathogenesis of this bacteria has not yet been clarified, but recent molecular reports have shown some advance in understanding it. Host colonization by uropathogens occurs in the lumen of the bladder within the stratified epithelium [24]. PA has an arsenal of extracellular products, such as pyocyanin, exotoxin A, and elastase, that damage the host tissue and impair the immune system response [25]. PA is a versatile pathogen known for its cytotoxic and invasive capabilities. Recent scientific evidence has shown that it can exhibit both intracellular and extracellular behaviors [24,26]. However, there is extensive evidence that PA preferentially binds to, invades, and injures wounded epithelium [27,28,29]. When uroepithelium is infected or traumatized, the superficial layers undergo exfoliation. As a result, the deeper layers are exposed, and may subsequently become colonized; it is within these underlying epithelial layers that uropathogens may persist in a quiescent state [24,30]. The ability of PA to survive intracellularly may play a significant role in contributing to the chronicity and recurrence of urinary tract infection [24]. Interestingly, in humans, it has been shown that *PA* UTIs result in higher readmission rates compared to UTIs caused by other uropathogens, underscoring the clinical importance of this problem [31].

PA can adapt to hostile environments within hosts by secreting a variety of virulence factors that contribute to successful infection and disease manifestation. For instance, it contains lipopolysaccharide (LPS), which protects the external leaflet and damages host cells [32]. It can promote tissue damage, facilitate attachment, and enhance recognition by host receptors. LPS has also been implicated in antibiotic tolerance and biofilm formation [33]. The outer membrane proteins (OMPs) play a significant role in nutrient exchange, adhesion, and antibiotic resistance [34].

The production of flagella and type IV pili facilitates adherence to the host and is responsible for motility through the urinary tract, enabling its colonization of the bladder and kidneys [25]. A possesses secretion systems that are vital for colonization of the host, adhesion, swimming, and swarming in response to chemotactic signals [35]. Hemolysin production enhances the ability to cause kidney infection [36].

Biofilm, a structured community of bacteria encased in a protective extracellular matrix, is a crucial virulence factor in PA. Biofilm formation by PA on catheters or within the bladder can enhance resistance to host immune responses and antibiotic treatment, making infections difficult to eradicate. Numerous studies in both human and veterinary medicine have reported that PA with biofilms exhibit resistance to various biocides, such as quaternary ammonium compounds, chlorine-based disinfectants, aldehydes such as glutaraldehyde, triclosan, benzalkonium chloride, chlorhexidine, hydrogen peroxide-based disinfectants, and 2-phenoxyethanol [15,37]. Quorum sensing systems are also crucial, as they enable PA to form biofilms and coordinate its behavior based on population density [38]. The pathogenesis of PA consists in its ability to adhere and colonize, form biofilms, produce virulence factors, evade immune responses, and remain in the host tissue [35].

### 3.2. The Opportunistic Behavior

The urinary tracts of dogs and cats exhibit anatomical and physiological differences that influence their susceptibility to PA infection. In dogs, the longer and narrower male urethra, along with the presence of the prostate gland, provides additional barriers to bacterial colonization, whereas the shorter female urethra facilitates bacterial ascension, increasing the risk of UTI. In cats, the male urethra is also narrow, but the prevalence of UTIs is lower due to the unique composition of the feline urobiome and the lower incidence of underlying predisposing conditions compared to dogs [39]. Significant physiological differences include urine concentration. Cats have an extraordinary ability to concentrate urine, an adaptive trait that reduces urine volume and may limit bacterial proliferation. However, this also increases the risk of crystalluria and urethral obstruction [40].

In humans, the composition of the urobiome and personal hygiene practices play a critical role in preventing urinary infections. Additionally, close contact with pets may act as a vector for MDR pathogens [41]. Studies indicate that the pathogenesis of PA in the urinary tract is closely associated with its ability to form biofilms, a characteristic observed in both humans and animals [42]. However, cats appear to mount a more effective immune response against initial PA infections, which may account for their lower incidence compared to dogs. In contrast, the predisposition of dogs to chronic conditions such as diabetes and kidney disease may contribute to their higher rates of PA infection [41].

Dogs and cats can acquire PA infections through environmental exposure (contaminated water, soil, surfaces), inhalation, or animal bites [25,43]. However, nosocomial infections are more common, reflecting their opportunistic nature. PA requires compromised epithelial barriers to invade and proliferate [44]. This explains the increased risk in immunocompromised animals, those with trauma, tumors, catheters, prolonged immobilization, and recurrent UTIs [45,46,47]. 

In humans, the presence of patients in hospitals for more than 30 days increases the risk of acquiring a UTI by almost 100% [48]. Although, in a veterinary oncology study, PA was isolated in 11% of patients, but only 3.6% were implicated in UTIs [49]. Broad-spectrum antibiotic use (cephalosporins, aminoglycosides) is a risk factor for MDR PA [30]. While PA accounts for 10–15% of human nosocomial infection, comparable data in veterinary medicine are lacking [18,50,51]. On the other hand, a study involving 32 canines with renal disease found that PA was the most frequently isolated microorganism in culture [52].

PA is a significant uropathogen in catheterized patients due to its ability to adhere to catheter surfaces and form robust biofilms which facilitate its establishment and persistence in the urinary tract [18,53]. On the other hand, urinary catheters disrupt the bladder’s natural defenses, increasing susceptibility to bacterial colonization and ascending infection [5,18,54]. Bacteriuria in catheterized animals is notably high (10% to 55%), but the prevalence of PA in these cases is unknown [55,56]. A recent study by Ataya et al. [46] demonstrated a low incidence of PA in these patients but a high prevalence of PA MDR strains.

### 3.3. Antimicrobial Resistance

Once established on host tissue, PA employs a combination of intrinsic, acquired, and adaptive resistance mechanisms to survive and resist antibiotics (see Table 1) [15,57].

Intrinsic mechanisms include outer membrane permeability, which can be reduced by modulating porins, and the production of antibiotic-inactivating enzymes such as β-lactamases and aminoglycoside-modifying enzymes. Additionally, the efflux system plays a key role in removing harmful agents from within the bacterial cell, with the overexpression of efflux pumps being associated with MDR.

Acquired mechanisms involve genetic mutations or the acquisition of new genetic material through processes such as transduction, conjugation, and transformation, often influenced by exposure to antibiotics.

Finally, adaptive mechanisms allow PA to increase its resistance to antibiotics, contributing to persistent infections. Among these mechanisms, persistent cells, which are metabolically inactive and have a reversible phenotype, and the formation of biofilms, which facilitate the exchange of genetic material and protect bacteria from antibiotic treatment and disinfectants, stand out. Drug resistance associated with biofilm formation is linked to structures such as flagella, pili, and other adhesins [58]. Furthermore, the quorum sensing system enables PA to coordinate behaviors and form biofilms efficiently, especially in chronic and biofilm-associated infections. These mechanisms contribute to the high resistance of PA to a wide range of antibiotics, including β-lactams, aminoglycosides, fluoroquinolones, and colistin.

Recent studies have shown that prior use of antimicrobials promotes the emergence of resistant pathogens. This happens because the antimicrobial used causes selective pressure in all susceptible bacteria that are killed or inhibited, while resistant ones survive. As a result, the composition of the host’s microbiome is altered, and the proportion of resistant bacteria may increase and become the majority in the host. This explains why the emergence of multidrug-resistant strains is more common in hospital environments or communities with uncontrolled use of these drugs [59]. Thus, Lin et al. [60] found resistance levels of PA in otitis and pyoderma in dogs to be lower than in other studies, attributing this to the fact that the animals studied had no history of prior antimicrobial use. On the other hand, Vingopoulou et al. [61] observed that resistance to fluoroquinolones was significantly higher in dogs that had been treated systemically with antimicrobials up to 6 months before the study.

**Table 1:** Resistance mechanisms of PA.

PA Resistance Mechanisms			
Mechanisms		Associated Antibiotic	References
Intrinsic Natural mechanisms to eliminate antimicrobials	Outer membrane (OM) permeability: decreases OM permeability by managing the number of non-specific porins present in the membrane. Antibiotic-inactivating enzymes: produce enzymes capable of selectively inactivating or modifying antibiotics (ex:β-lactamases and aminoglycoside-modifying enzymes). Efflux system: Remove harmful agents from the interior of the bacterial cell. The overexpression of multiple efflux pumps by this species has been associated with multidrug resistance.	β-lactams, Aminoglycosides, Fluoroquinolones	[15,17,62,63]
Acquired Implies a change in the microorganism’s genetic material.	Gene mutation Acquisition of new DNA (transduction (by bacteriophage); conjugation (cell–cell contact); transformation (transfer of naked DNA). (It is influenced by external stressors, such as exposure to antibiotics.)	β-lactams, Aminoglycosides, Fluoroquinolones, Colistin	[15,62,64]
Adaptative Increases their ability to resist the effects of antibiotics and contributes to persistent infections.	Persistent cells: metabolically inactive cells that exhibit tolerance to antibiotics and possess a transient, reversible phenotype. They arise under stressful conditions and are implicated in chronic bacterial infections as well as the recurrence of biofilm-associated infections Biofilm: A structured community of bacteria embedded within a self-produced extracellular polymeric substance matrix. It can colonize natural tissues or inert materials. Facilitates the exchange of genetic material among bacteria. Quorum sensing: Enables PA to form biofilms and coordinate behavior based on population density.	Increased tolerance to antibiotic and disinfectant treatments(>1000-fold).	[15,54,65,66,67,68]

## 4. Transmission

The transmission of antimicrobial-resistant bacteria has become a One Health issue as MDR bacteria can be transmitted between humans, animals, and the environment [59,69].

**Figure 1:**
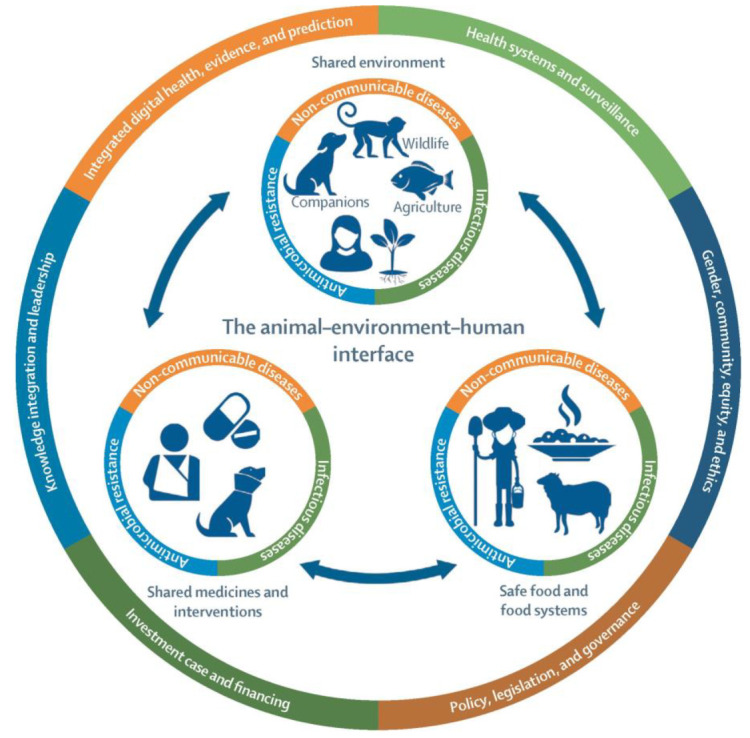
The importance of the One Health approach [91].

It is a fact that the use of antimicrobials in animals and humans promotes the development of MDR bacteria in both commensal and pathogenic bacteria [70]. In the environment, soil itself acts as a natural reservoir for antimicrobial-resistant bacteria; additionally, manure-treated soil and animal and human fecal waste contribute to the maintenance and the spread of MDR bacteria [71]. Furthermore, soils and irrigation water are sources of contamination for vegetables and fruits, where resistant bacteria have been identified [70]. Transmission by food consumption or wildlife vectors can also occur [59]. Thus, the impact of MDR bacteria (including MDR PA) is a problem that should be addressed with a One Health approach (Figure 1). In hospital environments, where antimicrobial pressure is higher, there is an increased likelihood of encountering MDR bacteria. In this context, the transmission of these pathogens can easily occur between hospital settings and the community [59]. The impact of this transmission is becoming increasingly relevant in clinical practice, especially as more people bring their pets closer to them.

Because of this approximation, MDR bacteria can be transmitted directly through close interactions between pets and humans; or indirectly through environmental transmission, where bacteria can spread when humans and pets coexist in the same household [21,52,72,73]. A study conducted by Jin et al. [69] demonstrated that AMR can be transmitted from animals to humans and vice versa, with evidence indicating that this transmission has risen over the past two decades. However, the authors note that most studies offer a low level of evidence regarding this transmission, highlighting the need for more robust studies.

Regarding PA, the information is lacking, but some studies and case reports deserve some consideration. Evidence indicates that some species, such as sheep, chinchillas, rabbits, and snakes, can be naturally infected by this microorganism [74,75,76,77]. Additionally, wildlife species also exhibit some sensitivity to PA [78,79,80]. The capacity of these species to act as reservoirs for PA increases the potential for the transmission of this pathogen [81,82]. According to Płókarz D. and Rypuła K. [41] dogs and cats are significant reservoirs for PA and can transmit it to humans through saliva, aerosols, urine, feces, and close contact. On the other hand, molecular studies have shown that clones associated with human infections have also been identified in animal infections; however, this does not serve as definitive evidence of transmission between species. Consequently, information regarding this transmission is still limited, but reports indicate the need for careful practices when PA is present [83,84].

Although there is no evidence of animal-to-animal transmission, the transmission between susceptible individuals (with cystic fibrosis) has been documented in humans [12,85]. Mohan et al. [21] described a case of PA respiratory infection transmitted from a human to a cat. Close contact between the individuals likely facilitated transmission, potentially via droplet spread, aerosolization, or indirect contact with contaminated surfaces [12]. The transmission of PA from animals to humans is not yet clearly understood [86], but a possible case of transmission from a dog to a young child with CF has been reported by Michl et al. [87]. Morris et al. also [88] described cross-contamination between the environment and owners and dogs with PA otitis. These studies were case reports or case series studies, suggesting that more rigorous primary studies, such as longitudinal, cohort, or case–control studies are needed to provide higher levels of evidence [69]. However, it is important to emphasize that the veterinary niche represents a potential reservoir of resistance genes for pathogenic bacteria in both animals and humans [84].

Most PA infections are primarily acquired from environmental sources rather than through direct transmission from infected individuals [12]. A study in human medicine demonstrated that environmental contamination, rather than cross-transmission, was the predominant source of infection in most cases [89]. The ability of PA to thrive in various potential niches and adapt to different hosts is concerning. This adaptability may enhance their capacity to acquire new traits related to both virulence and resistance [90]. These findings highlight the impact of this pathogen on human, animal, and environmental health. Because of this, a One Health approach could be used to address this pathogen.

The spread of MDR bacteria can occur not only within but also between hospitals [47,92,93]. Because of this, when a PA infection occurs, it is crucial to consider all potential environments, including veterinary hospitals and homes [43,47,93]. In a Swiss study, the percentage of MDR bacteria found in clinics for companion animals was detected at 8.2% of the sampling sites [93].

To prevent the transmission of these bacteria, efforts should focus not only on reducing the use of antimicrobials, but also on establishing barriers to effectively prevent environmental contamination [90]. This can be achieved through the implementation of improved sanitation measures to contain resistant strains within healthcare settings and animal production facilities [90]. For instance, veterinary clinics with poor infection and prevention control standards showed an extensive environmental contamination [69]. The WHO has published guidelines for the “Prevention and Control of Carbapenem-Resistant Enterobacteriaceae, *Acinetobacter baumannii*, and PA in Health Care Facilities”, which can be easily consulted, and which provide valuable guidance for clinical implementation [94].

## 5. Prevalence/Epidemiological Data

### 5.1. Europe

**Figure 2:**
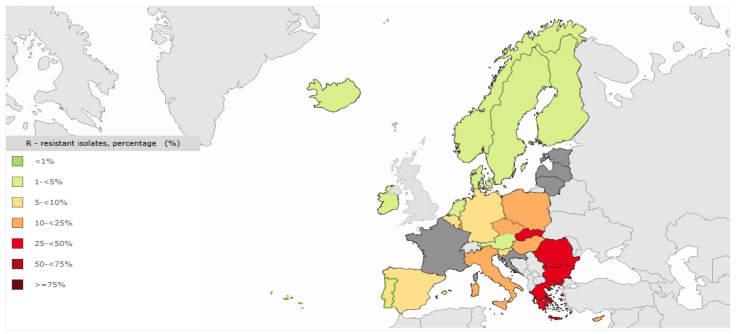
The distribution of MRD PA across various countries in the European Union. https://atlas.ecdc.europa.eu/public/index.aspx accessed on 30 January 2025.

In the EU, antibiotic resistance among PA is a pressing concern, with significant implications for public health. According to The European Antimicrobial Resistance Surveillance Network (EARS-Net), the incidence of MDR PA is lower compared to other Gram-negative bacteria; however, it remains a leading cause of healthcare-associated infections across Europe [95] (Figure 2). This issue can be partly attributed to shared risk factors, including high consumption rates of broad-spectrum antimicrobials and varied infection prevention and control (IPC) practices in healthcare settings.

A notable resistance gradient is observed across the EU, with a clear north-to-south and west-to-east pattern. The northern and western regions generally report lower resistance rates, while the eastern and southern regions show higher rates of antimicrobial resistance (Figure 1). In particular, carbapenem resistance in PA is notably more prevalent in these regions [95].

Between 2019 and 2023, the estimated incidence of PA infections resistant to piperacillin-tazobactam, ceftazidime, and carbapenems has shown significant increases, reflecting a troubling upward trend in resistance. By 2023, the highest estimated incidence of PA infections in the EU was attributed to carbapenem resistance, followed by resistance to piperacillin-tazobactam, fluoroquinolones, ceftazidime, and aminoglycosides [95].

The WHO classified carbapenem-resistant PA as a high-priority pathogen, as these agents are often utilized as last-line therapies in humans [96]. In the EU, the distribution of carbapenem resistance in PA is variable; some countries report resistance rates exceeding 50%, while others have rates below 5% [96].

In 2022, data from the European Surveillance of Antimicrobial Consumption Network (ESAC-Net) showed that 17 out of 28 countries met or exceeded the WHO’s target of 60% for total antibiotic use. However, only 4 out of 15 countries that reported reached this target [97].

The slow progress towards EU targets on antimicrobial consumption, coupled with the continued rise in the use of ’Reserve’ and broad-spectrum antibiotics, underscores the urgent need to strengthen efforts aimed at curbing unnecessary and inappropriate antimicrobial use across all sectors of healthcare.

### 5.2. Portugal

**Figure 3:**
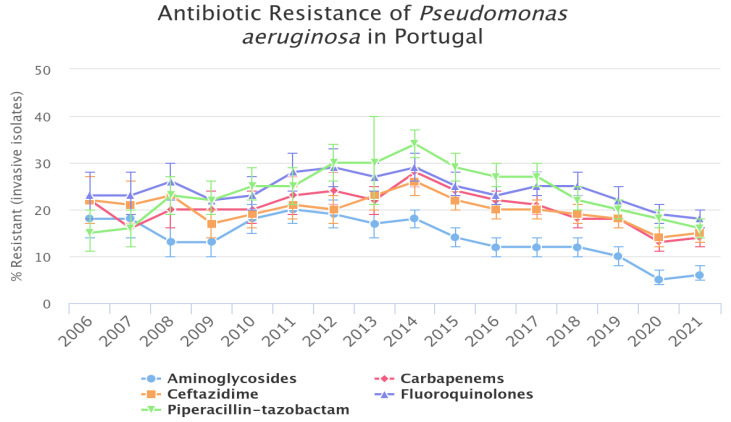
Antibiotic resistance of Pseudomonas in Portugal. OneHealthTrust. ResistanceMap: antibiotic resistance of *Pseudomonas aeruginosa* in Portugal. 2025. OneHealthTrust. Date accessed: 28 January 2025.

Portugal has reported resistance rates to the main classes of antimicrobials exceeding 10%, although there has been a declining trend over the years (Figure 3). However, this decline has not been observed in the group of multidrug-resistant bacteria.

Resistance to PA in Portugal gradually increased until around 2014–2016, after which there has been a steady decrease in resistance levels (Figure 3). Interestingly, data from the European Medicines Agency (EMA) indicated that antibiotic sales in Portugal fluctuated during this period, peaking in 2016 [98]. The veterinary sales percentage ranged from 70% in 2011 to 55% in 2021. Among the antibiotics, fluoroquinolones and piperacillin-tazobactam showed the highest levels of resistance by PA in Portugal. Since 2011, the EMA has reported a significant overall decrease of 51.5% in annual antibiotics sales, including a 25% reduction in third- and fourth-generation cephalosporins, a 36.9% decline in fluoroquinolone sales, and a remarkable 76.5% reduction in polymyxin sales [98].

The EMA attributes these declines in antibiotic sales to various factors, including changes in legislation, the implementation of electronic prescriptions in January 2022, and workshops and training sessions for veterinarians and producers. It is possible that the reduction in resistance rates of PA may be connected to these decreases in antibiotic sales [98].

### 5.3. Animals with UTI

The prevalence of PA in UTIs in cats and dogs is unclear as of yet, and varies with geographic location, the presence of underlying conditions, and prior antibiotic use [46]. In contrast, in humans, it is well documented and is responsible for 2.4–9% of UTIs [38]. Table 2 shows the various published studies on PA in dogs with UTIs around the world.

In the United States, studies conducted across various states have reported prevalence rates ranging from 1.3% in Louisiana (2012–2014) to 3.6% in California (1969–1995), with a higher prevalence observed in female dogs and the Rottweiler breed [45,99]. However, in Illinois (2019–2020), the prevalence was 2.99%, with low resistance rates to gentamicin, piperacillin-tazobactam, amikacin, and imipenem [100].

In Europe, the prevalence of PA also varies significantly. In Italy (2013–2015), the rate was 13.4%, with gentamicin identified as the most effective antimicrobial [101]. In Portugal (1999–2016), the prevalence was only 2% [102] while in Germany (2019–2020), it was 2.1%, with 33% of isolates classified as MDR. Larger European studies conducted between 2013 and 2018 reported prevalences ranging from 3% to 4%, with high resistance levels to fluoroquinolones such as enrofloxacin and marbofloxacin [103].

In Asia, a study conducted in Chiang Mai, Thailand (2012–2016), reported a prevalence of 13.1% [4]. In Hong Kong (2018–2020), the prevalence was 4.9%, with high resistance levels to antimicrobials such as amoxicillin, ampicillin, and trimethoprim-sulfamethoxazole, while susceptibility to ciprofloxacin and ofloxacin was below 25% [9].

In Brazil (2021–2022), the prevalence was 2%, with high resistance rates to penicillin, ceftriaxone, and trimethoprim-sulfamethoxazole [104]. Conversely, in Egypt (2024), prevalence reached 12.5%, with all feline isolates classified as MDR, showing susceptibility only to fluoroquinolones and nitrofurantoin [105].

These variations can be attributed to factors such as the geographic differences, study periods, data collection methodologies, and canine populations evaluated. Furthermore, the antimicrobial resistance of PA is a growing concern in veterinary medicine. Studies indicate that this bacterium is often resistant to multiple antibiotics, including fluoroquinolones and aminoglycosides, complicating the treatment of infections in dogs [106].

In cats, the data also reveal significant variations between countries. Table 3 shows the various published studies on PA from catswith UTIs around the world.

In Australia, among the 107 samples analyzed, the prevalence was 1.6%, with resistance documented in 5 of the 14 antimicrobials tested. Susceptibility was observed for marbofloxacin and ciprofloxacin, while enrofloxacin showed intermediate results [107].

In Italy (2013–2015), the prevalence was 5.5%, with gentamicin being the most effective antimicrobial, and a documented increase in resistance over time [101]. In Spain (2016–2018), 5.2% of cats tested were positive for PA, with 4.6% being multidrug-resistant, and 0.2% XDR. Furthermore, one isolate was classified as pan-drug-resistant (PDR), with high levels of resistance (≥50%) to antimicrobials such as amoxicillin-clavulanate, ampicillin, and first- to third-generation cephalosporins [1].

In Southeast Asia, in Chiang Mai, Thailand (2012–2016), the prevalence was significantly higher (25%), with all isolates classified as MDR [4]. These data are alarming, as they demonstrate a growing global trend of resistance in regions where the control of antimicrobial use and hygiene practices may be less rigorous, exacerbating the problem of bacterial resistance. In Portugal, data indicate a prevalence of 4% (1999–2016) and 1.6% (2017–2021), reinforcing the importance of local surveillance [108]. In Hong Kong (2018–2020), the prevalence was 4.8%, with 90% of isolates showing resistance to cefovecin and reduced susceptibility to fluoroquinolones and nitrofurantoin [9].

In Germany (2019–2020), 4.1% of the analyzed cats presented PA, with 41.7% MDR and four strains classified as XDR [3]. In Brazil (2021–2022), the prevalence was 2%, with high rates of resistance to trimethoprim-sulfamethoxazole, penicillin, and ceftriaxone [104]. Finally, European studies from 2013 to 2018 indicated prevalences ranging from 0% to 4.2%, with high rates of resistance to enrofloxacin and marbofloxacin [103]. Resistance to these classes of antibiotics is of particular concern because these drugs are frequently used to treat bacterial infections in animals and humans.

**Table 2:** Published studies on PA in dogs with UTIs around the world.

Country and Year	No. of Dogs and %PA Isolates		Resistance	Notes	References
California (EUA) 1969–1995	N = 2165	%PA = 3.6%		>Frequency of PA in dog females and Rottweiler breed	[45]
Italy 2013–2015	N = 253	%PA = 13.4%	GEN is the most effective	An increase in antimicrobial resistance over time	[101]
Louisiana (EUA) 2012–2014	N = 208	%PA = 1.3%			[99]
Spain 2016–2018	N = 52	%PA = 3.8%	≥50%—AMC, AMP, LEX, CEF, CXM, CTX, CVN, ENR, PRA DOX, FOF, NIT, SXT	In total, 4% of isolates were MDR	[1]
Chiang Mai (Thailand) 2012–2016	N = 203	%PA = 13.1%			[4]
Portugal 1999–2016	N = 700	%PA = 2%			[102]
Ilinois (EUA) 2019–2020	N = 803	%PA = 2.99%	Low resistance to GENT, TZP, AMK, IPM		[100]
Central Italy 2020	N = 450	%PA = 3.55%	AMP, NIT, TET, SXT, CPP, FQs	Susceptibility: AMK, DOX; GENT, IMI	[109]
	93.7% MDR				
Germany 2019–2020	N = 697	%PA = 2.1%	ENR PRA	33% MDR	[3]
Hong Kong 2018–2020	N = 2011	%PA = 4.9%	AMX, AMC, AMP, CFE, CVN, CPD, CRO, CET, DOX, NIT, SXT <25%: CIP, ENR, MAR, OFX		[9]
Brazil 2021–2022	N = 90	%PA = 2%	SXT, PEN, CRO, CTX		[104]
Romania 2022–2023	N = 83	%PA = 4%	CXM NIT		[110]
Europe 2013–2014	N = 606	%PA = 3%	1GC, TET, CVN, SXT.	Extremely high resistance levels against ENR and MAR	[103]
Europe 2017–2018	N = 773	%PA = 4%			
Cairo (Egypt) 2024	N = 146	%PA = 12.5%	AMX, 3GC, 4GC, DOX, SXT	Susceptibility: FQS and NIT 100% PA isolated from feline were MDR strains	[105]

1GC: first-generation cephalosporins; 3GC: third-generation cephalosporins; 4GC: fourth-generation cephalosporins; AMC: amoxicillin clavulanic acid; AMP: ampicillin; AMK: amikacin; AMX: amoxicillin; CEF: ceftiofur; CPP: cephalosporins; CET: cefalotin; CIP: ciprofloxacin; CPD: cefpodoxime; CRO: ceftriaxone; CTX: cefotaxime; CVN: cefovecin; CXM: cefuroxime (axetil or sodium); DOX: doxycycline; ENR: enrofloxacin; FOF: fosfomycin; FQS: fluoroquinolones; GEN: gentamicin;IMI: imipenem; LEX: cephalexin; MAR: marbofloxacin; NIT: nitrofurantoin; OFX: ofloxacin; PEN: penicillin; PRA: pradofloxacin; SXT: trimethoprim–sulfamethoxazole; TET: tetracycline; TZP: piperacillin–tazobactam.

**Table 3:** Published studies on PA in cats with UTIs around the world.

Country and Year	No. of Cats and %PA Isolates		Resistance	Notes	References
Austrália	N = 107	%PA = 1.6%	In total, 5/14 antimicrobials tested	Susceptible: MAR and CIP Intermediate: ENR	[107]
Italy 2013–2015	N = 81	%PA = 5.5%	GEN is the most effective	An increase in antimicrobial resistance over time	[101]
Spain 2016–2018	N = 24	%PA = 5.2%	≥50%—AMC, AMP, LEX, CEF, CXM, CTX, CVN, ENR, PRA DOX, FOF, NIT, SXT	In total, 4% of isolates were MDR and one isolate from a cat was PDR	[1]
	4.6% MDR 0.2% XDR				
Chiang Mai (Thailand) 2012–2016	N = 49	%PA = 25%		All isolates were MDR	[4]
Portugal 1999–2016	N = 240	%PA = 4%			[102]
Central Italy 2020	N = 185	%PA = 6.48%	AMP, NIT, TET, SXT, CPP, FQs	Susceptibility: AMK, DOX; GENT, IMI	[109]
	100%MD				
Germany 2019–2020	N = 313	%PA = 4.1%	ENR PRA	41.7% MDR 4 XDR e 1PDR	[3]
Hong Kong 2018–2020	N = 3708	%PA = 4.8%	AMX, AMC, AMP, CFE, CVN, CPD, CRO, CET, DOX, NIT, SXT <25%: CIP, ENR, MAR, OFX	In cats, 90% were resistant to CVN	[9]
Portugal 2017–2021	N = 5306	%PA = 1.6%			[108]
Brazil 2021–2022	N = 90	%PA = 2%	SXT, PEN, CRO, CTX		[104]
Romania 2022–2023	N = 83	%PA = 0%			[110]
European 2013–2014	N = 263	%PA = 0%	1GC, TET, CVN, SXT	Extremely high resistance levels against ENR and MAR	[103]
European 2017–2018	N = 379	%PA = 4.2%			

1GC: first-generation cephalosporins;; AMC: amoxicillin clavulanic acid; AMP: ampicillin; AMK: amikacin; AMX: amoxicillin; CFE: ceftiofur; CPP: cephalosporins; CET: cefalotin; CIP: ciprofloxacin; CPD: cefpodoxime; CRO: ceftriaxone; CTX: cefotaxime; CVN: cefovecin; CXM: cefuroxime (axetil or sodium); DOX: doxycycline; ENR: enrofloxacin; FOF: fosfomycin; FQS: fluoroquinolones; GEN: gentamicin;IMI: imipenem; LEX: cephalexin; MAR: marbofloxacin; NIT: nitrofurantoin; OFX: ofloxacin; PEN: penicillin; PRA: pradofloxacin; SXT: trimethoprim–sulfamethoxazole; TET: tetracycline.

## 6. Diagnosis

Diagnosis is based on a combination of clinical signs and standard bacterial culture (gold standard) to identify the pathogen [111]. Furthermore, while traditional culture takes 24–72 h, rapid methods like matrix-assisted laser desorption/ionization time-of-flight mass spectrometry (MALDI-TOF MS) and genomic analysis provide results within minutes to an hour, enabling timely treatment [112,113]. Antimicrobial susceptibility testing (AST), including MIC and disk diffusion methods, is crucial for choosing the best treatment [114]. Biofilm-associated infections, particularly those caused by PA, can yield negative culture results due to bacterial aggregation within the biofilm matrix [115,116]. Newer molecular techniques like 16S rRNA PCR, LAMP, FISH, and CLSM offer promising alternatives for detecting biofilm-associated pathogens [116,117]. 

During cystotomy for urolithiasis, bladder mucosal biopsies should be cultured alongside urolith analysis to identify potential uropathogens missed by standard urine cultures [118]. PCR testing can characterize bacteria and detect resistance genes, aiding antimicrobial selection [119,120]. However, this approach is not routinely used for PA.

## 7. Treatment

The treatment should always be guided by culture and susceptibility testing; however, initial empirical treatment may be necessary. For uncomplicated UTIs, the International Society for Companion Animal Infectious Diseases (ISCAID) guidelines recommend amoxicillin/amoxicillin-clavulanic acid or trimethoprim-sulfamethoxazole as first-line options [5]. In PA infections, due to its intrinsic resistance mechanisms, only third-generation cephalosporins, fluoroquinolones, or aminoglycosides are considered suitable for UTI treatment [3]. While carbapenems are effective against severe nosocomial PA infections, their use should be reserved for human medicine [121]. Aminoglycoside use in UTIs is limited by nephrotoxicity, parenteral administration requirements, reduced effectiveness in acidic urine, and inactivation by purulent debris [122]. For complicated UTIs, nitrofurantoin, fluoroquinolones, and third-generation cephalosporins may be reasonable first-line choices. In pyelonephritis, veterinary fluoroquinolones or cefpodoxime are considered appropriate [5]. In catheterized animals, systemic antibiotics are recommended, and catheter removal or replacement should be considered prior to initiating therapy. Inadequate management of UTIs, especially in catheterized animals, increases the risk of bacteremia [50].

Some studies have investigated antimicrobial combinations or novel agents to address the challenges in treating PA, particularly those involving biofilms. Saini et al. [66] demonstrated the potential efficacy of azithromycin combined with ciprofloxacin in treating biofilm-associated urinary tract infections. Novel antibiotics such as doripenem (a carbapenem) and plazomicin (a next-generation aminoglycoside) may offer future therapeutic options [123].

Treatment success can be challenging, and recurrent infections following treatment cessation have been reported [124]. However, data on the specific rate of treatment failure in dogs and cats is limited [12].

### Alternative Therapeutic Strategies

Excessive antibiotic use contributes to the emergence and dissemination of MDR organisms, including PA [123]. This necessitates the exploration and development of alternative therapeutic strategies. Several promising approaches are summarized in Table 4. However, the translation of many of these innovative strategies into clinical practice remains limited due to concerns regarding cost, potential side effects, and overall safety [123].

The analysis of alternative therapeutic approaches for the treatment of PA reveals a diverse array of strategies, each with varying levels of efficacy according to the reviewed studies. The AMP2041 peptide has shown promising results against MDR strains in both humans and animals, with inhibition rates exceeding 80% in some studies [125]. Fruit extracts, such as those from *Cornus mas* L. and *Sorbus aucuparia*, have demonstrated a significant reduction in bacterial growth, with an efficacy of approximately 60–70% in UTIs in companion animals. Nanoparticles have excelled in reducing biofilm formation, achieving success rates above 70% [110,126].

Phillyrin, a quorum sensing inhibitor, has exhibited up to an 80% reduction in pathogen virulence in experimental models [127]. Phage therapy has shown exceptionally high efficacy, with up to 90% success in eradicating skin infections in dogs models [60,128]. Protein Epitope Mimics, while promising, still require further evaluation, showing an efficacy of around 60–70% in inhibiting resistant strains. Although vaccines against PA are not yet widely available, experimental vaccines have demonstrated up to 70% efficacy in preventing infections [129,130]. Additionally, the use of electrochemical structures to combat biofilms and enhance antibiotic penetration has proven effective, achieving success rates of 75–80% [131].

Alternative strategies for the treatment of PA reveal promising advances in combating infections caused by this MDR pathogen. Despite differences in reported efficacy rates, which range between 60% and 90% depending on the approach and experimental context, these results highlight the importance of exploring alternatives to traditional antibiotic-based therapies. However, there is still a need for additional studies to validate the clinical applicability of these strategies on a large scale and in different epidemiological scenarios.

## 8. Discussion

UTIs are among the most prevalent medical conditions affecting domestic cats and dogs. These infections not only pose significant health risks, but also contribute to the emergence and proliferation of antibiotic resistance [46].

While there is extensive literature on the antimicrobial resistance patterns of PA in human medicine, data for this in veterinary medicine have been poorly characterized, particularly in UTIs [9]. These findings may be explained by the greater impact of PA on human health compared to that on small animals. For instance, this pathogen has been associated with high morbidity and a substantial mortality rate in human medicine, accounting for 3.57 million deaths in 2019 [16].

PA is frequently isolated from ear and wound swabs in dogs and from the nasal cavity in cats [22]; this may explain the limited number of studies focusing on the prevalence of PA in the urinary tract in these species.

With this review, we aim to provide insights into the prevalence of urinary tract infections in dogs and cats, as well as how this has changed over the years. However, due to the aforementioned reasons, the data available for clarification are still limited. Nonetheless, we believe that, given its impact on public health, further investigation into this topic should not be overlooked. Table 2 and Table 3 present a compilation of studies from around the world that document the isolation of PA in UTIs in dogs and cats, respectively. The prevalence of PA varies over the years and across different countries in these studies. However, when compared to other pathogen isolates, the prevalence of PA remains low in both species (varies between 1.3% and 13.4% in dogs and 0 and 25% in cats). These findings may be attributed to the opportunistic nature of PA, which only affects a small group of patients (immunocompromised animals). Furthermore, the bacterium’s ability to form biofilms may lead to an underestimation of its presence in culture results, potentially resulting in false negatives [116]. This underscores the need for further research to enhance our understanding of the epidemiology of PA in UTIs among small animals. We believe that understanding the prevalence of PA among pets is crucial from both veterinary and human medicine perspectives [83].

European studies from 2013 to 2018 indicated prevalences ranging from 0% to 4.2% in cats and 3–4% in dogs (Table 2 and Table 3). According to Figure 2, in 2023 the percentage of MDR PA varies greatly from country to country, ranging from 1% to 50%.

*E. coli* is the most isolated pathogen in UTIs [5,6,132] in contrast, a study conducted in Chiang Mai reported PA as the predominant isolate in feline UTIs (25% of the isolates) [4]. This discrepancy between the microbial profiles observed in Thailand and those in other countries may be attributable to variations in empirical antimicrobial prescription practices, which can create selective pressure on bacterial populations, or can be justified by natural geographical differences [7].

The number of dogs with suspected UTIs was higher than that of cats (Table 2 and Table 3). This observation may be attributed to a greater number of dogs visiting veterinary clinics or the ease of obtaining samples from canines [108]. These findings are also consistent with numerous studies that report a higher incidence of non-infection urinary tract diseases (idiopathic and inflammatory) in cats [133,134]. In fact, according to Rodriguez [135] and Heseltine [134], less than 10% of cats with urinary clinical signs have a positive urine culture. In a study by to Płókarz D. and Rypuła K. [41] dogs presented higher frequencies of PA (92%) than cats (72%), but these data considered samples from the nasal cavity, ear, and wounds. Perhaps cats are more resistant to PA than dogs, which could explain the differences observed between the two species regarding this bacterium in these reports. But more studies are needed to determine this.

In the context of UTI, sufficient data are not yet available. The data regarding UTIS in dogs and cats cannot be compared based on the information provided in Table 2 and Table 3. This is primarily due to the significant differences in the population sizes of cats and dogs included in the respective studies, which may influence the observed prevalence rates. Therefore, without more balanced data or additional studies specifically targeting PA prevalence in UTIs among both species, it remains unclear whether the trend observed in other samples would hold true for UTIs [30]. On the other hand, different methods were employed to assess antibiotic susceptibility, and the years of the studies also varied. These factors are crucial for the interpretation of results, as the guidelines for interpretation PA sensitivity have undergone changes. In humans, the elderly and women appear to be more susceptible to PA UTI. In companion animals, bacterial UTIs are also more common in females, and age is a significant risk factor as well. In a previous study, Norris et al. [45] found a higher frequency of PA in female dogs and in the Rottweiler breed (Table 2) [136].

Some studies [103,137] in Table 2 and Table 3 indicated a gradual increase in the prevalence of MDR pathogens over the years [3,14]. If we compare with EARS-NET data, we can observe a similar trend. A significant isolation of MDR PA strains was observed across all the studies. Included in this, Gómez et al. [13] and Darwich et al. [1] identified PA as one of the bacterial species with the highest levels of MDR strains. These findings underscore the versatility of PA, attributed to its diverse resistance mechanisms, the presence of numerous virulence factors, and its capacity to form biofilms [14]. At same time, these findings have enhanced the overuse and misuse of antibiotics over the years and the need to improve antimicrobial prescription procedures [138,139]. Likewise, the EMA and ESAC-Net report a high consumption of antibiotics in recent years in Europe.

The prevalence of AMR strains was found to be higher in cats compared to dogs [13]. Similar results were also obtained by Gómez-Beltránthe authors of [13]. Although there are currently no publications addressing this disparity, it is plausible that differences in the urobiome composition between these species may contribute to this observation. Additionally, the nature of urinary diseases in cats, which are often inflammatory, may facilitate the growth of MDR PA in the urinary tract. Further studies are necessary to elucidate these relationships and to better understand the underlying factors contributing to the observed resistance patterns in this bacterium. Likewise, PDR strains were isolated exclusively from feline species in Spain and in Germany. These results are frightening, as in these cases clinicians did not have any effective antimicrobials to eliminate this pathogen [3].

Damborg et al. [43] propose that pet-associated bacterial zoonoses represent a relatively neglected area of study compared with food borne zoonoses, and we support this assertion due to the lack of information available on the topic of PA in companion animals. While the precise transmission routes of PA are not fully understood, some studies suggest potential transmission between humans, animals, and the environment. Therefore, a One Health approach (Figure 1) is crucial for effectively addressing these infections [14]. Robust biosecurity and infection control measures are essential for combating the spread of PA, particularly in hospital environments [140]. However, controlling PA presents significant challenges due to its inherent resistance to certain biocides and its ability to adapt and survive in challenging environments [15].

The innate resistance of PA contributes to its reduced susceptibility to beta-lactams, cephalosporins, trimethoprim, and sulfonamides [1,103]. As shown in Table 2 and Table 3, the levels of resistance to these antimicrobials were consistently high across all studies. Additionally, elevated resistance levels were also observed for tetracyclines and cefovecin [1,103]. The resistance patterns to fluoroquinolones and aminoglycosides displayed significant variability among the studies. This variation may be attributed to factors such as geographical location, differences in prescription practices, or the specific populations studied [105].

According to ISCAID guidelines, nitrofurantoin, fluoroquinolones, and third-generation cephalosporins may be reasonable first-line choices for complicated or resistant UTIs [5]. However, these studies revealed a wide range of resistance patterns for these antibiotics. For instance, fluoroquinolones demonstrated efficacy in populations in Europe, Illinois, and Egypt, but were ineffective in a feline population in Germany. Gentamicin exhibited consistent efficacy across species and locations in most studies, likely due to careful dosing practices in veterinary settings to minimize toxicity [109]. These findings suggest geographical consistency in PA susceptibility patterns [103]. Notably, high resistance rates to marbofloxacin and enrofloxacin were observed in European studies, potentially attributed to the widespread use of fluoroquinolones in these species, particularly for UTIs [141,142,143]. Likewise, the data reported by EARS-Net show the highest incidence of PA infections in the EU which was attributed to carbapenem resistance, followed by piperacillin-tazobactam, and fluoroquinolones. Although PA resistance to carbapenems was generally low in companion animal studies, they were not evaluated in all studies. Because of this, the antimicrobial profiles of *PA* isolates are difficult to compare between different studies [83]. But in the Illinois study, some isolates were found to be resistant to imipenem [83,100]. In Europe, the eastern and southern regions show higher rates of carbapenem antimicrobial resistance. It may be interesting to introduce this drug in future studies in these countries.

It is important to note that the in vitro susceptibility results may not always accurately predict the clinical outcomes. Some antibiotics achieve significantly higher concentrations in urine compared to plasma, which can enhance their efficacy in treating UTIs despite in vitro resistance [111]. Understanding in vivo sensitivity could clarify treatment failure rates for this versatile pathogen. In human medicine, the relationship between multidrug resistance and clinical outcomes also remains unclear [144].

The optimal treatment approach should be guided by culture and AST results [139,141]. However, in clinical practice, constraints such as time, financial limitations, and physical resources can impede the implementation of this standard [139]. Consequently, clinicians may resort to empirical antimicrobial therapy. While necessary in some cases, this practice can contribute to the emergence and spread of antimicrobial resistance [101]. A study conducted in an Italian veterinary teaching hospital found that only 5% of antimicrobial prescriptions were based on microbiological culture and susceptibility testing [138]. Additionally, Robbins et al. [141] and Mouiche et al. [142] reported a substantial number of cases where antimicrobials were prescribed unnecessarily. Additionally, data from ESAC-NET reveal that antimicrobial consumption in most EU countries exceeds the target set by the WHO. These results highlight the need for an antimicrobial control system.

Inadequate regulatory oversight and ineffective surveillance of antibiotic prescriptions contribute to the emergence and spread of MDR in pets with UTIs [145]. For example, the implementation of an online antimicrobial stewardship program in Switzerland significantly reduced antibiotic prescriptions for UTIs in dogs and cats [146,147]. Urgent action is needed to improve the surveillance and monitoring of antimicrobial resistance in veterinary medicine [145,148]. We believe that effective antimicrobial stewardship strategies include educational initiatives, clinical guidelines, and computer-based decision support systems [141,145].

Due to this paradigm, future treatments may focus less on eradicating pathogens and more on maintaining the integrity of the urinary tract barrier [30]. Concurrently, research is actively exploring alternative strategies for eliminating pathogens, as summarized in Table 4. While these approaches offer promising potential, further research is necessary to validate their efficacy and safety. We hope that this review serves as a foundation for future studies aimed at investigating the prevalence of PA in UTIs, improving monitoring techniques, and developing effective strategies for its elimination.

## 9. Conclusions

Current understanding of PA prevalence and its resistance patterns in canine and feline UTIs is limited. Existing research suggests a low overall prevalence of PA in UTIs, but the incidence of MDR strains appears to be alarmingly high. Further investigation is crucial, particularly regarding feline UTIs. PA antimicrobial resistance patterns varied geographically and temporally. Therefore, the empirical antimicrobial agents suggested by the ISCAID guidelines are not always effective against isolated strains. This highlights the importance of bacterial culture. which should be performed whenever UTIs are suspected to minimize antimicrobial overuse. Future research can include the correlation of MDR strains with clinical outcomes in order to clarify their impact on treatment success. Future studies should also consider more epidemiological data on animals with PA infection. Genomics has proven to be a powerful tool for understanding PA and guiding targeted antibiotic therapy. The exploration of alternative therapies to antibiotics can also be a useful tool to minimize their impact. In any case, a collaborative effort (human, animal, and environment) is essential to eliminate this pathogen (a One Health approach).

Implementing antimicrobial stewardship programs is increasingly urgent, and research like this can contribute to enhanced monitoring efforts.

## Figures and Tables

**Table 4:** Published research on alternative strategies for antimicrobial treatment.

Alternative Strategy	Description	References
Peptide Amp 2041	Positive results in human and animal research.	[125]
Fruit extracts (*Cornus mas* L. and *Sorbus aucuparia*)	Might be used as substitutes or adjuvants for antibiotics in UTI isolates of companion animals.	[110]
Nanoparticles	Promising in reducing PA growth as well as in the formation of its biofilm.	[126]
Phillyrin	It is an effective inhibitor of quorum sensing with potential for PA infection therapy.	[127]
Phages	In a study on dogs with PA skin infection, the application of phages was greatly effective in the control of this pathogen.	[60,128]
PEM (protein epitope mimetic)	It has emerged as a novel class of antibiotics against PA. They inhibit the transport of LPS to the outer membrane.	[123]
Vaccines	There is not a PA vaccine currently available on the market, although several different vaccines and several monoclonal antibodies have been developed in recent decades.	[129,130]
Electrochemical scaffold	Disruption of bacterial biofilms and increase in antibiotic penetration	[131]

## Data Availability

Not applicable.
